# Welfare and Performance of Three Turkey Breeds—Comparison between Infrared Beak Treatment and Natural Beak Abrasion by Pecking on a Screed Grinding Wheel

**DOI:** 10.3390/ani11082395

**Published:** 2021-08-13

**Authors:** Stefanie Grün, Klaus Damme, Matthias Müller, Marie Franziska Sommer, Paul Schmidt, Michael Erhard, Shana Bergmann

**Affiliations:** 1 Department of Poultry and Education, Bavarian State Research Center for Agriculture, Mainbernheimer Straße 101, 97318 Kitzingen, Germany; klaus.damme@baysg.bayern.de; 2 National Institute of Animal Health, Bavarian Health and Food Safety Authority, Eggenreuther Weg 43, 91058 Erlangen, Germany; matthias.mueller@lgl.bayern.de (M.M.); MarieFranziska.Sommer@lgl.bayern.de (M.F.S.); 3 Statistical Consulting for Science and Research, Große Seestraße 8, 13086 Berlin, Germany; paul.schmidt.mail@gmail.com; 4 Chair of Animal Welfare, Ethology, Animal Hygiene and Animal Husbandry, Department of Veterinary Sciences, Faculty of Veterinary Medicine, LMU Munich, Veterinärstraße 13/R, 80539 Munich, Germany; m.erhard@tierhyg.vetmed.uni-muenchen.de

**Keywords:** animal welfare, Auburn, beak trimming, blunting, B.U.T. 6, B.U.T. Premium, poultry

## Abstract

**Simple Summary:**

Beak trimming of turkeys is an animal welfare issue. It can result in acute pain, potential chronic pain, and a change in feeding, drinking, and pecking ability and general behavior. It is still permitted by law in Germany when the intervention is necessary to protect turkeys from feather pecking and cannibalism. In the present study, an alternative method using grinding wheels (blunting disks) that were fitted in the feed pans when the turkeys were six weeks old was tested. The disks were expected to blunt the beak tips during feeding and reduce the severity of pecking injuries. Six hundred male turkeys of three breeds (B.U.T. 6, B.U.T. Premium, Auburn) were housed separately in 24 groups. The birds in 12 groups were beak trimmed, those in the other 12 were not, but received the blunting disk. The results showed a noticeable beak abrasion in birds provided with blunting disk. Injuries and plumage conditions were equivalent between the treatments. Summarized, the blunting method may be an alternative to infrared beak treatment, but its effectiveness should be confirmed under commercial conditions. The blunting method could potentially result in improved animal welfare of turkeys by minimizing acute pain, chronic pain, and injurious pecking.

**Abstract:**

Feather pecking and cannibalism are behavioral disorders that cause animal-welfare-relevant and economic problems. To mitigate these problems, the beaks of conventionally reared turkeys are usually already trimmed in the hatcheries. To find an alternative to beak trimming, we conducted this study with male turkeys of three breeds: B.U.T. 6, B.U.T. Premium and, Auburn (200 turkeys per breed). Half of the birds had infrared-trimmed beaks; the other half had intact beaks. For each treatment combination (breed, beak status), 25 turkeys were housed in one section. A screed grinding wheel was installed in each feed pan of the non-beak-trimmed turkeys as of week six to facilitate natural beak abrasion until slaughter. Eight randomly selected turkeys per section were regularly examined to record injuries, plumage condition, and beak dimensions. In addition, 96 beaks from randomly slaughtered birds were examined macroscopically and histologically. The results concerning injuries and plumage condition showed in most cases no differences between the beak-trimmed turkeys and the ones provided with the blunting disks. The histological examinations revealed alterations in only the beak-trimmed birds. We can conclude that the blunting method smoothens the beak during feeding and thus may be a possible alternative to beak trimming.

## 1. Introduction

Beak trimming for the prevention of feather pecking and cannibalism has been a long-time topic of discussion, not only in Germany. In July 2015, the Central Association of the German Poultry Industry, Berlin (German designation: Zentralverband der Deutschen Geflügelwirtschaft e. V.) and the German Poultry Producers Association, Berlin (Verband Deutscher Putenerzeuger e. V.) came to terms with the Federal Ministry of Food and Agriculture, Bonn (Bundesministerium für Ernährung und Landwirtschaft) in a voluntary agreement on the abandonment of beak trimming [1], initially for layer hens. Since 1 August 2016, the abandonment has been in force, and since 1 January 2017, beak-trimmed layer hens have no longer been housed in Germany. For turkeys, a comparable decision was also discussed, and the abandonment was scheduled for 1 January 2019 for female turkeys. A regulation regarding male turkeys was supposed to follow. In the meantime, research focusing on the omission of beak trimming was intended to find a solution to the problem of severe injuries that intact beaks can cause during fattening [1]. However, to date, no satisfactory results are available, so the beaks of conventionally reared turkeys in Germany are still trimmed by infrared beam on the first day of life (DOL) in the hatchery. According to the German Animal Welfare Act § 6 Section 1, “the complete or partial amputation of body parts or the complete or partial removal or mutilation of organs or tissues of a vertebrate is prohibited” [2]. However, an exception permit is stipulated in § 6 Section 3 of the German Animal Welfare Act: The prohibition does not apply when a “case relates to § 5 Section 3 No. 2 to 6 and the procedure in the individual case of intended use of the animal is essential for its protection or for the protection of other animals (…)”. At the European level, according to Article 24 No. 2 of the Council of Europe’s recommendations for the keeping of turkeys [3], “procedures on turkeys are strictly prohibited.” However, also in this case, the text following this stipulation formulates an exception permit stating that the beak of turkey chicks under the age of 10 days may be trimmed if measures for environmental enrichment and improved management do not suffice to prevent severe injuries among the birds. Furthermore, the exception permit stipulates that at most one third of the upper beak (measured from the tip of the beak to the nares) or of the upper and lower beak may be trimmed within the first 10 DOL. Beak trimming after the 10th DOL is only possible with veterinary indication and if permitted by national law [3]. In organic turkey fattening in Germany, beak trimming has been prohibited since 5 September 2008 according to the implementation rules of Order (EG) No. 889/2008. Nevertheless, farmers fattening organic turkeys (in Germany, mostly female B.U.T. 6 or Kelly Bronze) also had partial problems with feather pecking and cannibalism [4], although the requirements in organic animal husbandry were much higher than those in conventional rearing.

The German poultry industry has been obliged to allow infrared beak trimming only on the first DOL in the hatchery [1]. In addition, the German hatcheries agreed in July 2015 to use the infrared method as temporary bridging technology only [1]. This method has the advantage that it is performed simultaneously with other procedures (sexing, vaccination) in the hatchery, and thus additional handling-related stress can be prevented. The method does not cause open wounds and is easily standardized [5]. In studies on laying hens where the beaks were trimmed with a hot blade, alterations on the beak tip and the bill tip organ were found [6,7,8,9]. These changes at the beak tip (sensory feedback was reduced) are often associated with pain and possibly even with chronic pain [6,7,8,10]. Gentle [11] differentiates between acute pain and chronic pain. Acute pain lasts for seconds to days and follows nociceptive stimulation or minor trauma; it vanishes after healing. This acute pain is also inflicted during beak trimming and lasts for a few hours afterwards [11]. In contrast, chronic pain lasts for weeks or even years and is seen in chronic disease states or after major trauma. In addition, changes in behavior can often be observed in connection with chronic pain [11]. Studies on behavioral changes after beak treatment showed that, e.g., infrared beak-trimmed birds spent less time drinking and feeding than untrimmed birds [10]. Gentle [11] described the development of chronic pain only in chicks that were beak trimmed at older ages. Typically, the hot-blade devices are used at an older age, e.g., the 7th or 10th DOL. So, a comparison with infrared beak trimming is always influenced by this age effect. As different studies with chickens describe [12,13], the age at which the animals are beak trimmed has a critical influence on the beaks. For example, Gentle and Keegan [12] showed that the upper beaks were significantly shortened in animals that were beak trimmed (with a hot-blade debeaker) on the 7th or 10th DOL, even after the 42nd DOL, compared with animals that were beak trimmed as day-old chicks (with hot-blade or with infrared treatment). Furthermore, Hughes and Gentle [13] described neuroanatomical changes mainly in older chicks that were beak trimmed. Another important influencing factor is the severity of the treatment. For infrared beak treatment, Struthers et al. [14] showed that guide-plate sizes, lamp power, mirror design and beak exposure time play an important role. Localized clew-like alterations of the nerve fibers and a blunted bone tip were noticed in turkey chicks examined after the infrared beam procedure [5]. In our own preliminary study [15], non-beak-trimmed B.U.T. 6 male turkeys provided with two types of blunting disks in the feed pan were compared with beak-trimmed and with non-beak-trimmed turkeys without an integrated blunting disk. The histological examinations of infrared-trimmed turkey beaks (after the 119th and 147th DOL) showed irregular bone alterations and neuronal proliferations, indicating an amputation neuroma [15]. Nonetheless, beak trimming still seems indispensable. Krautwald-Junghanns et al. [16] reported that beak trimming reduced the prevalence of severe skin injuries. In their study on the incidence of skin injuries in beak-trimmed turkeys, the head and the neck area were most frequently affected by pecking injuries and cannibalism. There are also numerous studies showing that beak-trimmed poults have significantly fewer injuries, less feather loss and lower mortality rates than non-beak-trimmed poults [13,17].

The objective of the present study was to further examine blunting as an alternative method to beak trimming, and thus as a way to improve the welfare of turkeys. Blunting is a process that facilitates natural beak abrasion, which means to blunt the sharp tip of the beak to reduce injurious pecking. In nature, turkeys generally forage the ground for food. They are persevering foragers and frequently peck at rough surfaces such as sand, soil or rocks [18], and this behavior causes abrasion of the overlapping beak tip. In this study, we installed a screed grinding wheel as a rough substrate in the feed pan to imitate the natural process of beak blunting during feeding. The blunting method was previously tested on layer hens [19] and, slightly modified, on Japanese quails [20]. Taskin and Camci [20] installed a pumice stone in the bird section and found a significant beak abrasion and a more intact plumage compared with quails from a control section. A significant beak abrasion occurred when the abrasive surface material was integrated in the feed pan [19]. However, the authors were not able to show an impact of the blunting on injuries or plumage condition [19]. In our own preliminary study [15], we also found beak abrasion due to the blunting disks without significant differences in plumage condition or injuries as compared with the beak-trimmed turkeys without the integrated blunting disk. Based on the results of the preliminary study [15], the present study focuses on using a 30-grit screed grinding wheel as a blunting disk. We compared two treatments (infrared beak treatment and natural beak abrasion by pecking on a screed grinding wheel as a blunting disk) and three turkey breeds (B.U.T. 6: heavy-weight turkeys with white plumage; B.U.T. Premium: moderate-weight turkeys with white plumage; Auburn: light-weight turkeys with black plumage) to identify animal-welfare-relevant differences.

## 2. Materials and Methods

This study was performed according to the legal regulations of the German Animal Welfare Act, 2006 (German designation: Tierschutzgesetz—TierSchG) [2], the German Order on the Protection of Animals and the Keeping of Production Animals, 2006 (Tierschutz-Nutztierhaltungsverordnung—TierSchNutztV) [21], the German Order on the Protection of Animals during Transportation, 2009 (Tierschutztransportverordnung—TierSchTrV) [22] and the German Order on the Protection of Animals in Connection with Slaughter and Killing, 2012 (Tierschutz-Schlachtverordnung—TierSchlV) [23].

### 2.1. Rearing Phase

#### 2.1.1. Animals and Experimental Setup

The open-barn research unit of the Bavarian State Research Center for Agriculture, Department of Poultry and Education in Kitzingen (GPS coordinates: 49°44′2.69″ N, 10°8′50.56″ E; 206.5 km in a straight, direct line from Kitzingen to Munich), Germany, has a total length of 37 m and a total width of 10 m. The poultry house has a concrete floor, concrete side walls and sandwich panels on the roof. In total, 821 one-day-old turkey chicks of the breeding company Moorgut Kartzfehn von Kameke GmbH & Co. KG, Bösel, Germany, were housed on 26 July 2018 (average outdoor temperature: 28 °C, relative humidity: 59%). The chicks were 209 B.U.T. 6 male turkeys, 207 B.U.T. Premium male turkeys, 207 Auburn male turkeys and 198 Auburn female turkeys. Both B.U.T. 6 and B.U.T. Premium male turkeys have white plumage. In Germany, B.U.T. 6 male turkeys in conventional rearing are usually fattened as heavy-weight carving turkeys and reach a slaughter weight of about 21.33 kg at an age of 20 weeks [24]. The breed B.U.T. Premium is considered a moderate-weight carving turkey with the male turkeys reaching an average slaughter weight of 19.87 kg in the 20th week of life [24]. In the Auburn breed, the male turkeys have black, and the female turkeys have brown plumage. They are light-weight turkeys that are used for organic turkey fattening in Germany. Male Auburn turkeys have an average slaughter weight of 14.27 kg in the 20th week of life [24].

**Figure 1:**
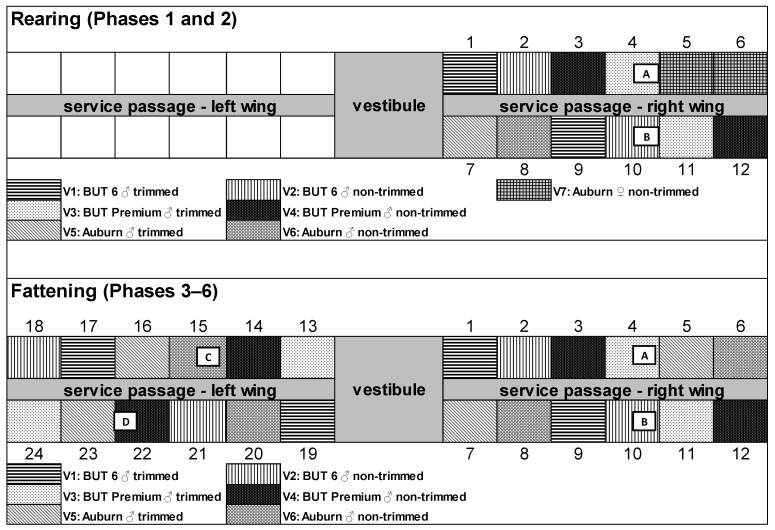
Experimental setup showing the distribution of the three breeds in the barn sections (1 to 24) during rearing (=1st to 35th day of life; top) and fattening (=36th to 138th day of life; bottom). Only the right wing was used during rearing. A, B, C, and D indicate the locations of the data logger sensors that measured the temperature and relative humidity in the barn. B.U.T. = British United Turkey, V = variant.

At the hatchery, all chicks had been vaccinated against turkey rhinotracheitis. Half of the chicks of each breed had been beak-trimmed by infrared beam (Poultry Service Processor—PSP, Nova Tech Engineering LLC, Willmar, MN, USA). The settings of the PSP device for all groups were as follows: power 43, guard plate 24/20 as the smallest for turkeys; mirrors and other aids were not used. According to the manufacturer, the device calculates the radiation duration individually depending on the calibration. Female turkeys of the Auburn breed had to be taken on and were raised in the same barn (see housing scheme in Figure 1).

Due to limited rearing capacities, the Auburn breed had to be housed with more birds per square meter than the other breeds during the early rearing phase. Auburn turkeys were initially housed with 100 chicks per section, B.U.T. 6 and B.U.T. Premium turkeys with 50 chicks per section. The following groups (variants) were composed: V1 = beak-trimmed B.U.T. 6, V2 = non-beak-trimmed B.U.T. 6, V3 = beak-trimmed B.U.T. Premium, V4 = non-beak-trimmed B.U.T. Premium, V5 = beak-trimmed Auburn male turkeys, V6 = non-beak-trimmed Auburn male turkeys, V7 = non-beak-trimmed Auburn female turkeys. 

The research barn was divided into 24 experimental sections and two barn wings (Figure 1). Each section had a floor space of 10 m^2^. So, there were four replicates of three breeds of birds subjected to two beak treatments. Incoming airflow was automated via adjustable shutters, exhaust airflow via three ceiling fans, one energy-saving fan and two continuously adjustable fans for so-called vacuum ventilation. Rearing took place in the right wing (Sections 1 to 12). Each section was supplied with a corner barrier and a gas-fired infrared heater (TAS 41, Alke BV, BD Scherpenzel, The Netherlands) during the first 14 days, and with two drinking troughs (Plasson-Tränke MK II, Roxell^®®^, Hans Gaab, Wieseth, Germany), two feed pans (OPTIstart^TM^, Roxell^®®^, Hans Gaab, Wieseth, Germany) and additional feed on chick paper and egg carton during the entire rearing phase. Each section was littered down with 26 kg heat-treated, low-dust wood shavings; litter depth was 4 cm (PREMIUMSPAN^®®^, Hobelspanverarbeitung GmbH, Dittersdorf, Germany). Feeding during rearing took place in two phases (Table 1). Feed was purchased from the company BayWa AG FM/TH Franken 164 (Bamberg, Germany; for ingredients see Table A1). 

During the first 12 days, a step-down light program with a stepwise reduction in the light phase and intensity was applied. From the first to the seventh day, the light phase was reduced by one hour per day (from 24 h to 17 h per day), and afterwards, the light intensity was reduced (from between 80 and 100 lx to 20 lx) by means of a barn climate computer (FSUP 2e, Fancom B.V., Panningen, The Netherlands). The birds were vaccinated on the 15th DOL and in the 6th, 12^th^, and 18th week of life against Newcastle disease; on the 21st DOL and in the 8th week of life against turkey rhinotracheitis; and on the 28th DOL against hemorrhagic enteritis by staff of the Bavarian Animal Health Service (Tiergesundheitsdienst Bayern e.V., Poing, Germany). 

#### 2.1.2. Methods of Assessment

Biological Performance: At housing and after the first rearing phase (14th DOL), the body weight of all birds in each section was recorded. After Phase 2 (35th DOL), the birds were weighed again and randomly distributed to the 24 sections for fattening (25 birds per section; Figure 1).

Barn Climate Parameters: From the beginning of rearing, the following barn climate parameters were measured in regular intervals of about two weeks. The gaseous ammonia content was determined with a gas detector (MSA ALTAIR 2X, Mine Safety Appliances Company, Cranberry Township, PA, USA). The light intensity was recorded with a luxmeter (testo 435 Multifunktions-Messgerät, Testo SE & Co. KGaA, Lenzkirch, Germany); it was measured at animal head level at five locations in each section (elevated tier, below elevated tier, front feed pan, back feed pan, and next to drinking trough), always in six directions (top, bottom, north, south, west, and east). The temperature in degrees Celsius and the relative humidity in percent were recorded and saved hourly by four sensors of a data logger (Datenlogger LogBox-RHT, Art. Nr. 05680038, B+B Sensors, Donaueschingen, Germany) in the barn and one sensor outside of the barn (Figure 1); the data were called up after depopulation.

### 2.2. Fattening Phase

#### 2.2.1. Animals and Experimental Setup

**Figure 2:**
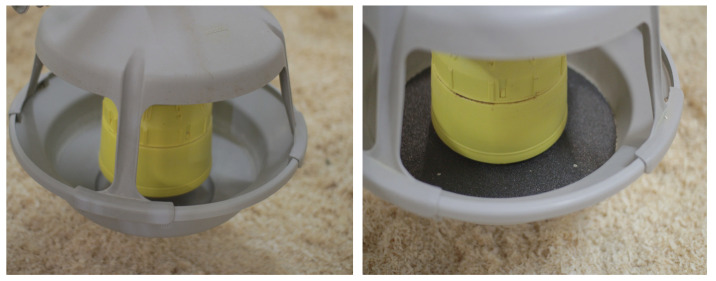
Feed pan without the blunting disk on the left side and with the integrated 30-grit screed grinding wheel on the right side.

Before the beginning of Phase 3 (62nd DOL), the feed pans for rearing were exchanged with feed pans for fattening (OPTIMAX^TM^, Roxell^®®^, Hans Gaab, Wieseth, Germany), and a 30-grit screed grinding wheel (company Spillner, Kitzingen, Germany) was installed as a blunting disk (Figure 2) in each feed pan for the 12 sections with non-beak-trimmed birds. The feeding space and drinking space per turkey were 12.06 cm and 12.57 cm, respectively.

In total, 600 turkeys were included in the experiment, and thus 25 turkeys were housed per section (targeted final stocking density: 49.06 kg/m^2^ for B.U.T. 6; 45.70 kg/m^2^ for B.U.T. Premium; and 35.11 kg/m^2^ for Auburn). The remaining male turkeys and the female Auburn turkeys did not take part in the study and were fattened separately. The previously occupied sections (1 to 12) were completely cleaned and freshly littered down with 26 kg soft-wood shavings before rehousing to create the same initial conditions in both barn wings. Once a week, 13 kg heat-treated, low-dust soft-wood shavings were added in each section. Furthermore, at the beginning of Phase 3, the elevated tiers, measuring 9600 cm^2^ (L: 2.40 m, W: 0.40 m, H: 0.50 m), were installed in each section, and lucerne briquets (Einstreuprofis, Partner der Landwirtschaft, Uwe Wagner, Seelingstädt, Germany) were provided as enrichment material. The drinking troughs were cleaned manually three times per week. The fattening phase consisted of four additional phases, during each of which phase-specific feed (of the company BayWa AG FM/TH Franken 164, Bamberg, Germany; for ingredients see Table A1) was provided (Table 1). This feeding phase interval was also used as interval for the animal examinations. 

#### 2.2.2. Methods of Assessment

Biological Performance and Animal Welfare Indicators: After Phase 3 (62nd DOL), Phase 4 (90th DOL), and Phase 5 (118th DOL), the birds were weighed in groups, the feed weight was adjusted in each section, and eight birds per section were randomly chosen and examined regarding the animal welfare indicators. We evaluated the plumage on the back, wings (Table A2) and tail (Table A3) and recorded injuries on the body (Table A4) and snood (Table A5) according to a modified hen scoring scheme of Niebuhr [25] ranging from Score 0 (no feather loss and no injuries) to Score 3 (high-grade injuries). The breast skin (breast buttons and breast blisters, Score 0 = no changes to Score 3 = profound changes; Table A6) was rated according to the scoring system of Straßmeier [26]. The foot pads were rated according to the five-score scheme of the Board of Trustees for Technology and Construction in Agriculture (Kuratorium für Technik und Bauwesen in der Landwirtschaft e. V.) [27] ranging from Score 0 (foot pad intact) to Score 4 (more than half of the middle foot pad injured, toes severely affected, or deep injuries); Table A7. The beak was assessed according to a four-score scheme [15], with Score 0 indicating a trimmed beak and Score 1 (no beak abrasion) to Score 3 (marked abrasion) relating to non-trimmed beaks; Table A8. In addition, the upper beak was measured. First, the upper-beak length (from anterior edge of the nares to beak tip) was measured with a flexible measuring tape. The overlap of the upper beak and after Phase 6 (137th DOL) that of the lower beak were measured with a digital caliper (Model SDK150, Globus Fachmärkte GmbH & Co. KG, Völklingen, Germany). After Phase 6, the birds were weighed individually, and eight birds per section were examined. In addition, at the end of Phase 6, all birds were examined regarding beak morphology (length of beak, upper-beak overlap, and lower-beak overlap in millimeters) and injuries of the snood according to the modified hen scoring scheme [25]. All losses, including the cause of death, were recorded. In addition, after Phases 3, 4, 5, and 6, fecal samples were taken from each variant and tested for endogenous parasites. 

Post-mortem Measurements: Both after Phase 5 and after Phase 6, two birds per section were randomly selected and slaughtered at the poultry slaughterhouse of the Bavarian State Research Center for Agriculture (Bayerische Landesanstalt für Landwirtschaft) in Kitzingen, Germany. The heads of the birds were vacuum preserved per variant and stored for 24 h at 2 °C for later histological examination at the Bavarian Health and Food Safety Authority (Bayerisches Landesamt für Gesundheit und Lebensmittelsicherheit, LGL) in Erlangen, Germany. The remaining birds were slaughtered at the turkey slaughterhouse “Süddeutsche Truthahn AG Handelsgesellschaft” in Ampfing, Germany. 

**Figure A1:**
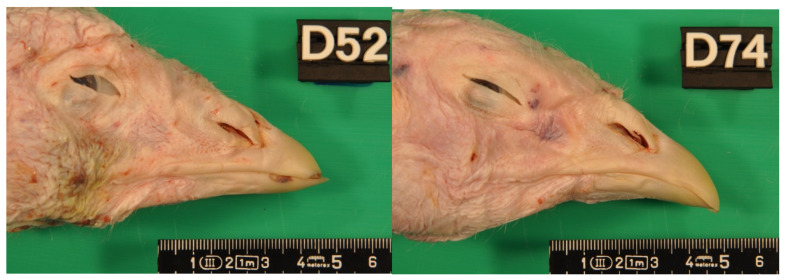
Macroscopic pictures of the heads of dead turkeys slaughtered after Phase 6 (138 day of life). On the left side (D52) is an infrared beak trimmed turkey and on the right side (D74) is the head of a turkey which had an integrated screed grinding wheel in the feed pan.

**Figure A2:**
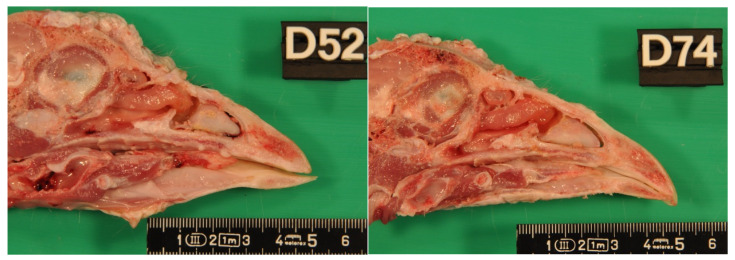
Macroscopic pictures of the turkey heads (also seen in Figure 1) which were split lengthwise with a band saw. On the left side (D52) is an infrared beak trimmed turkey and on the right side (D74) is the head of a turkey which had an integrated screed grinding wheel in the feed pan.

**Figure A3:**
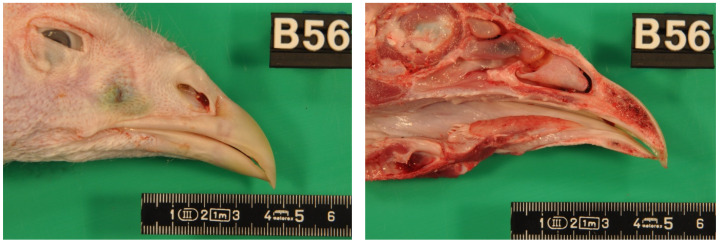
Macroscopic pictures of the head of a dead turkey of our preliminary study, slaughtered after Phase 6 (147 day of life) which was not beak-trimmed and without an integrated screed grinding wheel in the feed pan. On the left side the whole head can be seen and on the right side this head was split lengthwise with a band saw.

Histological Examination: First, the intact turkey heads were photographed laterally (Figure A1), and the upper beak lengths (measured from the anterior edge of the nares to the tip of the beak) as well as the overlap of the upper or lower beak were measured at the LGL in Erlangen. Second, the heads were split lengthwise with a band saw to measure the tip of the horny layer with a digital caliper (Figure A2).For a better comparability, a picture of a non-beak-trimmed turkey without integrated blunting disc, from our preliminary study, has been included in the appendix (Figure A3). One thin longitudinal section of each beak tip was excised for the histological examination. These samples were fixed in formalin (4% formaldehyde), decalcified, embedded in paraffin, thin-sectioned (4 μm), stained with hematoxylin–eosin and investigated microscopically by employees of the LGL (*n* = 1 thin section per beak tip). The main focus of the histological examination was to assess whether the normal physiological structures of the beak tip were preserved. The examined aspects included the morphology of the keratin sheath (rhamphotheca), the epidermis and the dermis with special emphasis on the nerve fibers and the presence of Herbst- and Grandry corpuscles and the praemaxillary bone. 

### 2.3. Statistical Analysis 

A sample size analysis was carried out during the planning of the experimental setup. All animal welfare indicators were measured on ordinal rating scales. Therefore, these variables were analyzed separately using cumulative logistic regression models for ordinal response variables. The experimental predictors age, breed and blunting as well as the interaction of the latter two were included as ordinary fixed effects. Of the climatic parameters, light intensity and gaseous ammonia content were also included as fixed effects, whereas temperature and humidity could not be included because of their high correlation with age. Section-specific effects were modeled as unstructured random effects for the intercept. The resulting hierarchical cumulative logistic regression models were estimated in a fully Bayesian approach using the Hamilton Monte Carlo approach implemented in the probabilistic programming language Stan [28] via the wrapper package brms [29] for the statistical programming language R (version 3.6.0, [30]). Results of these models were expressed as odds ratios (OR) as well as their corresponding standard errors and 95% credible intervals (CI). 

The continuous variables (biological performance data, upper-beak length, and upper-beak overlap) were analyzed with an SAS software program packet (version 9.4, SAS Institute Inc., Cary, NC, USA) according to a linear variance model (general linear model) with the fixed effects breed, blunting, and the interaction breed × blunting. Before the experiment, a *p*-value below 0.05 was defined as significant.

## 3. Results

### 3.1. Biological Performance

Compared with the other two breeds, Auburn showed less body weight (*p* < 0.001) just before slaughter (Table 2). Furthermore, the weight of B.U.T. Premium turkeys was lower (*p* = 0.009) than that of B.U.T. 6 turkeys after Phase 6. There were no differences in body weight (*p* = 0.420) after Phase 6 between beak-trimmed and non-beak-trimmed birds per breed. 

The mortality rate was 0.7% during rearing and 4.2% during fattening (of these losses, altogether 32% (eight birds) perished and 68% (17 birds) were culled during fattening). The following causes of death were differentiated: pecking injuries/cannibalism, heart failure, leg weakness/kick injuries, and other causes. Other causes included, for example, three birds that had died from unknown causes and thus were pathologically examined at the LGL in Erlangen, Germany. The pathological assessment revealed that two birds had a systemic infection with *Escherichia coli* and one bird had a moderate inflammation of a tendon sheath and joint. Because no generalized symptoms occurred in any of the flocks, no veterinary treatment (except vaccinations) was necessary during the whole experimental period. Four birds (Phase 3: one in V6; Phase 4: two in V4; Phase 5: one in V3) had to be taken out because of pecking injuries/cannibalism. Leg weakness with subsequent kick injuries caused by other birds was the main cause of death, especially at the end of fattening. None of the fecal sample screenings after each fattening phase revealed endogenous parasites.

### 3.2. Animal Welfare Indicators and Beak Morphology

Effects of the experimental and environmental predictors on animal welfare indicators are presented in Table 3 and Table 4. There was a positive correlation (OR = 1.03–1.06) between the age of the birds and the assessed parameters: with increasing age, the chance of higher assessment scores increased.

Regarding the examined welfare indicators, we could show that beak-trimmed Auburn turkeys had significantly fewer body injuries than beak-trimmed turkeys of the other two breeds (B.U.T. 6: OR = 0.30, CI = 0.09–0.77; B.U.T. Premium: OR = 6.72, CI = 1.94–16.89). In comparison, among the non-beak-trimmed turkeys, there was a significant difference between the Auburn and B.U.T. Premium turkeys (OR = 5.75, CI = 1.78–15.01). We noticed no significant difference in body injuries per breed between beak-trimmed and non-beak-trimmed turkeys with blunting disk (Table 4). 

The Auburn turkeys had significantly fewer snood injuries (22.0%) than the other two breeds (B.U.T. 6: 41.9% and B.U.T. Premium: 48.8%; Table 5). Furthermore, this variable showed a significant effect of beak trimming, with snood injury in 35.6% of the beak-trimmed and 48.2% of the non-beak-trimmed B.U.T. 6 turkeys (OR = 0.54, CI = 0.28–0.95), and similarly, snood injury in 41.2% of the beak-trimmed and 56.3% of the non-beak-trimmed B.U.T. Premium turkeys (OR = 0.44, CI = 0.22–0.76). A comparable result was not found in Auburn turkeys.

It was not possible to assess the variable plumage condition on the back and wings and the breast skin (for the variable breast blister) because of the small number of affected turkeys. For the variable breast button, we detected significantly (Table 4) fewer Auburn turkeys with alterations (on average 1.8%; Table 5) than B.U.T. Premium (12.8%) and B.U.T. 6 (17.2%) turkeys. The plumage assessment on the tail showed that Auburn turkeys had the fewest alterations and B.U.T. 6 turkeys the most. This variable showed no difference between beak-trimmed and non-beak-trimmed birds. For the analysis of the beak assessment, we considered only the non-beak-trimmed turkeys because all the beak-trimmed ones had Score 0. Although the B.U.T. Premium turkeys showed the strongest abrasion, we found no significant difference between the three breeds. The assessment of the foot pad health showed that the Auburn turkeys had significantly more foot pad alterations than the B.U.T. 6 and B.U.T. Premium turkeys (Table 4). Differences between B.U.T. 6 and B.U.T. Premium or between beak-trimmed and non-beak-trimmed birds were not significant (Table 4). Summarized, turkeys of the Auburn breed as compared with the other two breeds had fewer body injuries, plumage damages, snood alterations, and breast buttons, but they showed significantly poorer foot pad health. Furthermore, for the breeds B.U.T. 6 and B.U.T. Premium, we noticed fewer snood alterations in beak-trimmed than in non-beak-trimmed birds. After Phase 6, non-beak-trimmed B.U.T. Premium turkeys (30.0 mm) had a shorter upper beak than non-beak-trimmed B.U.T. 6 turkeys (31.3 mm), as well as the shortest upper-beak overlap (B.U.T. Premium: 1.44 mm vs. B.U.T. 6: 2.24 mm and Auburn: 2.68 mm; Table 6). The beak-trimmed turkeys hat no measurable positive overlap of the upper beak, and the non-beak-trimmed turkeys had no overlap of the lower beak.

### 3.3. Barn Climate Parameters 

The average gaseous ammonia content per phase was between 0 parts per million (ppm) in Phases 1 and 2 and 2.25 ppm in Phase 6. The average light intensity in the barn was 22 lx. The temperature, measured at the four predetermined locations in the barn (Figure 1), was on average 30.4 °C in the right barn wing in Phase 1. The temperature decreased continuously (on average 2.26 °C/week during rearing and 0.35 °C/week during fattening) down to 14.2 °C in the right and 13.1 °C in the left barn wing. The relative humidity at the beginning (Phase 1) was 51.4% in the right barn wing. It slowly increased to 75.6% in the right barn wing and 70.8% in the left barn wing in Phase 6. 

### 3.4. Post-Mortem Measurements 

At slaughter in Ampfing, Germany, rejections in each group were handled separately, so that total weight of the rejected parts and the reason for rejection could be recorded. The total rejections showed tendencies between the three breeds (B.U.T. 6: 33.9 kg; B.U.T. Premium: 22.6 kg; Auburn: 4.3 kg), but no clear trend considering beak-trimmed and non-beak-trimmed birds. Comparing total rejections of beak-trimmed birds (32.0 kg = 53%) with those of non-beak-trimmed birds with integrated blunting disk (28.8 kg = 47%) revealed a slight tendency in favor of non-beak-trimmed birds. Whole wings and wing tips were rejected most frequently (V1: 23 wings > V2: 18 wings > V3 and V4: 7 wings > V6: 3 wings > V5: 2 wings). In most cases, hematomas on the wings were the reason for rejection. Hematomas also had to be excised from other body parts. In some cases, the lower legs were cut off. Breast buttons (BBu) were another frequent reason for rejection (V3: 15 BBu > V2 and V4: 13 BBu > V1: 4 BBu > V6: 1 BBu > V5: 0 BBu).

### 3.5. Histological Examination

**Figure 3:**
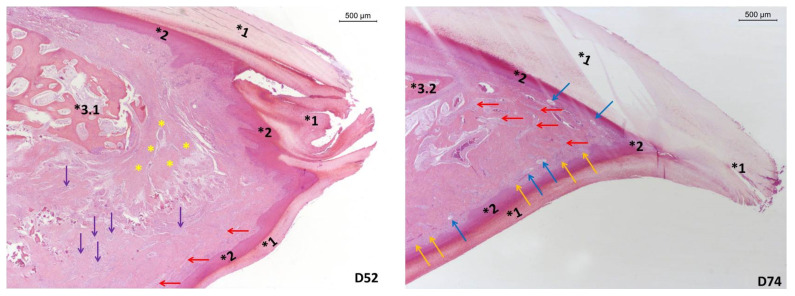
Histological examination of the upper beak tip after Phase 6 (138 DOL), 25× magnification and stained with hematoxylin-eosin. On the left side (D52) an infrared-trimmed beak tip and on the right side (D74) a beak tip of a turkey with integrated screed grinding wheel. *1 = keratin sheath (rhamphoteca); *2 = epidermis; *3.1 = blunted praemaxillary bone; *3.2 = praemaxillary bone; yellow asterisks = connective tissue in the form of scar tissue; purple arrows = disordered smaller nodes of nerve tissue; red arrows = nerve fibers; blue arrows = Herbst corpuscles; orange arrows = Grandry corpuscles.

The beak measurements of the slaughtered turkeys after Phases 5 and 6 are shown in Table 7 and Table 8. The histological examination of the beak tip specimens showed proliferations of nerve fibers, as well as disordered smaller nodes of nerve tissue, reduced Grandry corpuscles, and mostly missing Herbst corpuscles in all examined infrared beak-trimmed birds after Phases 5 and 6. Furthermore, bone tips seemed blunted, and connective tissue formed a type of scar tissue in all examined infrared beak-trimmed birds. In comparison the examination of non-beak-trimmed with the integrated blunting disk birds revealed a purulent inflammation of the beak tip of one B.U.T. 6 bird after Phase 5, which could not be ascribed to an abrasion by the grinding wheel. All other examined non-beak-trimmed birds with the integrated blunting disk (after Phases 5 and 6) showed no provable alterations (Figure 3).

## 4. Discussion

The weight of the turkeys after Phase 6 (weighing on the 137th DOL) differed according to breed but not according to beak condition (beak-trimmed vs. non-beak-trimmed with the blunting disk). These results are contrary to those of our preliminary study, in which the turkeys with integrated screed grinding wheel after Phase 6 weighed significantly less than the beak-trimmed turkeys without blunting [15]. In the preliminary study, we made sure that the turkeys with the blunting disk ate up all feed in the pans once a day for the last 11 fattening days. This was performed to achieve a higher abrasion effect on the beak. However, an associated reduced feed provision could possibly explain the weight difference. Damme and Urselmans [31], who tested the influence of various litter substrates and of blunting disks in the feed pan on biological performance parameters, also found no difference in the final fattening weight between beak-trimmed turkeys and non-beak-trimmed turkeys with a blunting disk. The live weights we recorded after the 20th week of life for all three breeds were above the listed weights [24].

The mortality rate of 0.7% during rearing and 4.2% during fattening in the present study can be considered low for male turkeys. In the preceding study with B.U.T 6 turkeys [15], in which two types of blunting disks were tested in comparison, the mortality rate during fattening was 12.5%. In the present study, the difference in mortality during fattening between beak-trimmed (4.0%) and non-beak-trimmed birds with screed grinding wheel (4.3%) is small. In comparison, Damme and Urselmans [31], who also used a screed grinding wheel and simultaneously tested various litter materials, found mortality rates of 10.5% in beak-trimmed turkeys and 16.6% in non-beak-trimmed turkeys with the blunting disk. In addition, our results showed a marked difference in mortality between the three breeds: mortality in Auburn turkeys was only 2.0% as compared with 5.0% in B.U.T. 6 and 5.5% in B.U.T. Premium turkeys. For conventional turkey fattening, cumulative mortality rates in weeks 5 to 20 were rated as follows: 10% as “average,” up to 5% as “good” and less than 3% as “very good” [18]. Elsewhere [32], an average mortality rate of 11.96% was reported for male turkeys without access to an outdoor-climate area in conventional turkey keeping. The Auburn turkeys from Aviagen are considered a robust strain with a low final fattening weight and are often used for extensive and organic turkey fattening, just like Kelly Bronze turkeys. During a study of a winter fattening phase, the total losses in the robust strain Kelly Bronze with 2.86% were markedly lower than the 11.1% evaluated in B.U.T. 6 turkeys [33]. However, when comparing mortality rates between the breeds of our study, we also have to consider that the stocking density (measured in kilograms per square meter) at the end of the study was markedly lower for the Auburn turkeys (35.11 kg/m^2^) than for the other two breeds (49.06 kg/m^2^ for B.U.T. 6, 45.70 kg/m^2^ for B.U.T. Premium). Kulke et al. [34] investigated two stocking densities (40 kg/m^2^ vs. 58 kg/m^2^) in non-beak-trimmed B.U.T. 6 male turkeys and found no differences considering mortality or stocking density. They only found a significant difference in the final fattening weight, with the birds fattened at lower stocking density showing a higher body weight at the end of fattening.

Furthermore, we noticed that the Auburn turkeys had fewer body injuries, snood injuries, breast buttons, and tail feather injuries than the turkeys of the other two breeds. Bergmann [33] also found overall significantly fewer plumage damages, breast buttons and injuries in a robust strain (Kelly Bronze) compared with the B.U.T. (Big) 6 breed. Another study compared the robust strain Kelly Bronze with the breed B.U.T. 6 and found overall fewer plumage damages and injuries in Kelly Bronze turkeys [26]. However, the Auburn turkeys had a significantly poorer foot pad health than those of the other two breeds in our study. This result is contrary to that of Mayne [35], who compared large white turkeys with Broad-Breasted Bronze turkeys and found that the white-feathered turkeys are more prone to foot pad dermatitis. Berk et al. [36] also investigated foot pad health in two breeds (B.U.T. 6 and Grelier Bronzés), comparing a heavy-weight breed with a so-called robust strain, but found no breed-related differences. The comparatively poor foot pad scores in the Auburn turkeys of the present study could be related to the rearing conditions. Owing to limited rearing capacities, the female Auburn turkeys had to be reared in the same barn as the males. Thus, the stocking density during Phases 1 and 2 for the male Auburn turkeys was twice as high as that for the male B.U.T. 6 and B.U.T. Premium turkeys. Previous studies reported that a higher stocking density, especially during rearing, is related to poor foot pad health [37,38]. The authors of those studies could show, on the one hand, that the foot pad skin especially during the first days of life is vulnerable to injuries and, on the other hand, that the litter moisture increases with increasing stocking density (birds per square meter) and thus the litter quality declines. These two aspects apply to our male Auburn turkeys during rearing. Other studies also reported poor litter quality as one of the main causes of foot pad dermatitis [35,36,39,40,41]. Foot pad health as a relevant parameter in the rearing phase was also evident in the study by Krautwald-Junghanns et al. [42], in which 45% of the turkeys showed epithelial necrosis already in the sixth week of life. Similarly, the turkeys in the study by Berk et al. [36] showed hyperkeratosis and superficial pododermatitis. Our foot pad assessment in the Auburn breed was complicated by the used assessment scheme [27], according to which foot pad lesions are scored as the area of darkly discolored, necrotic skin scales. This assessment is problematic because Auburn turkeys have relatively dark-pigmented feet. Thus, it was at times difficult to judge if dark colorations indicated a lesion or natural pigmentation.

In the present study, we found no significant difference in the assessed body injuries per breed between the non-beak-trimmed turkeys with blunting disk and the beak-trimmed turkeys. This finding, along with our results on beak morphology, suggests that the 30-grit grinding wheels in the feed pans caused a beak abrasion that was similarly effective in preventing pecking injuries as the infrared beak trimming. Several studies showed that beak-trimmed turkeys have fewer injuries and less feather loss than non-beak-trimmed turkeys [13,15,17]. Furthermore, at the end of the fattening period, Damme and Urselmans [31] found fewer pecked turkeys in the beak-trimmed group than in the non-beak-trimmed control group.

In the beak evaluation, we noticed for both the beak scoring and the measurements that the B.U.T. Premium turkeys tended to show the strongest abrasion, although beak abrasion also occurred in the other two breeds. Fiks-van Niekerk and Elson [19], who investigated beak abrasion in layer hens, also reported a significant beak abrasion. Furthermore, they found that blunting was most effective when the rough surface material is integrated in the feed pan and that the blunting method causes an effective beak abrasion. Various studies showed that turkeys occupy themselves for a longer time with provided enrichment materials if the material allows for feed intake while the turkeys are pecking at it, such as pecking stones that include grain [43,44,45]. The feed on top of the blunting disk ensures that the turkeys regularly peck at the disk and the beak smoothens naturally. Care must be taken that the feed filling level in the trough or feed pan is not too high so that the birds hit the blunting disk during pecking. To amplify this effect in our preliminary study, we made sure that the turkeys emptied the feed pan once a day during the last 11 days of fattening [15]. As a result, the turkeys had emptied the feed pans daily and increasingly pecked directly at the blunting disk. The integrated screed grinding wheels had a rough surface on both sides, so they can be used for two fattening phases. Owing to the material texture, it makes no sense to clean one side after use for reuse. Taskin and Camci [20] elaborated another approach to blunting in quail cages by installing a pumice stone at which the quails could peck and smoothen their beaks. Their study revealed not only significant beak abrasion but also a positive effect on the plumage of quails. In our preliminary study, we noticed no significant effect of blunting on the plumage condition [15]. However, in the present study, we detected that the turkeys (comparison non-beak-trimmed with blunting disk vs. infrared beak-trimmed) within a breed showed no significant differences in plumage condition. The lower-beak overlap in the infrared beak-trimmed birds of our study (measured after Phase 6; see Table 6) was on average 4.96 mm long. This value agrees with observations by Fiedler and König [5], who, after infrared beak trimming, frequently observed that the upper beak is shortened to a degree that the beak cannot be closed correctly and that the lower beak, due to lack of resistance and thus reduced abrasion, can grow into a shovel-like shape. In comparison, the upper-beak overlap in the herein examined non-beak-trimmed turkeys with a blunting disk was on average 2.12 mm long. Note that blunting disks were only installed in the feed pans of the non-beak-trimmed birds. It should be considered that, similar to the long upper-beak overlap in non-beak-trimmed turkeys, the lower-beak overlap could cause injuries during feather pecking or aggressive confrontations.

Regarding the assessed climate parameters, the gaseous ammonia content in the barn air was below 5 ppm during all measurements and thus always below the maximum permitted limit of 20 ppm [46]. The light intensity was ≥ 20 lx, meeting the requirements of the German Poultry Producers Association [46] (measured at eye level of the birds on three tiers that are arranged perpendicularly to each other). In the beak-trimmed turkeys of the present study, the histological beak examination revealed clew-like nerve fiber formations similar to those reported by Fiedler and König [5]. Furthermore, like in our preliminary study [15], we found irregular bone alterations and neuronal proliferations in all examined beak-trimmed turkeys. The pain perception of poultry is partly similar to that of mammals [11]. Therefore, one can assume that changes on the bone cause pain for the turkeys. Comparable alterations of the beak tip and the bill tip organ in beak-trimmed poultry have been described previously [6,7,8,9]. The development of neuromas with the presence of chronic pain was described as a consequence of hot-blade beak treatment [6,7,8]. However, second- and third-degree burns associated with chronic pain were also described with infrared beak treatment [5]. Other studies reported that infrared beak treatment of day-old chicks causes only acute pain but no chronic pain [10,11]. Finally, the reduced Grandry corpuscles and missing Herbst corpuscles, as found in the beak-trimmed turkeys of our study, can result in reduced sensory feedback and have an impact on animal welfare [10]. The turkeys that had an integrated screed grinding wheel in their feed pan showed no histological alterations of the bill tip organ or on the bone. These observations confirm the histological results of our preliminary study [15], which also showed no detectable negative alterations due to the blunting method.

## 5. Conclusions

Regarding the effectivity of the blunting disk, we could prove beak abrasion in all three breeds. Although B.U.T. Premium turkeys had the shortest upper-beak overlap by trend, they did not have fewer injuries than the Auburn or the B.U.T. 6 turkeys. Thus, considering the assessed variables (injuries on the body, plumage of the back and wings, tail feathers, except for snood injuries), the blunting method for a natural beak abrasion smoothing examined in this study was just as effective as beak trimming with the described infrared beam setting. Furthermore, the Auburn breed showed fewer injuries, plumage damages and rejections, but had poorer foot pad health than the turkeys of the other two breeds. Thus, we can conclude that the fattening of a robust turkey strain shows benefits in terms of animal welfare. Moreover, after correct application of the blunting disk, no pain, suffering, or damages due to this method were detected, neither macroscopically nor histologically. However, these findings are based on the investigation of one fattening phase with a limited number of animals and replicates. In summary, this blunting method represents an improvement in animal welfare and should be further investigated in practice.

## Figures and Tables

**Table 1:** Classification of rearing and fattening phases (P) and the feeding during rearing and fattening (feed from BayWa AG FM/TH Franken 164, Bamberg, Germany).

	Phase	Age		Feed	From	To
		Week of Life	Day of Life			
Rearing	P1	1–2	1–14	Gallugold PMK 1 GRAN 617001025, granulated	26 July 2018	08 August 2018
	P2	3–5	15–35	Bonimal GK PMK 2 PELL 6170820, pellets	09 August 2018	29 August 2018
Fattening	P3	6–9	36–62	Bonimal GK PMK 3 OG PELL 6171520, pellets	30 August 2018	25 September 2018
	P4	10–13	63–90	Bonimal GK PMK 4 OG PELL 6172220, pellets	26 September 2018	23 October 2018
	P5	14–17	91–118	Bonimal GK PMK 5 OG PELL 6173020, pellets	24 October 2018	20 November 2018
	P6	18–20	119–138	Bonimal GK PMK 6 OG PELL 6173820, pellets	21 November 2018	11 December 2018

**Table 2:** Average live weight and standard deviation (±) of male turkeys in grams (chick weight) and kilograms (all others) in relation to the variant, phase, and week of life.

Breed and Beak Treatment	Variant	Chick Weight (g) (*n* = 150)	After P1, 2nd LW (kg) (*n* = 615)	After P2, 5th LW (kg) (*n* = 615)	After P3, 9th LW (kg) (*n* = 595)	After P4, 13th LW (kg) (*n* = 589)	After P5, 17th LW (kg) (*n* = 580)	After P6, 20th LW (kg) (*n* = 523)
B.U.T. 6 trimmed	V1	62.04 ^ab^ ± 4.13	0.37 ^a^ ± 0.028	1.88 ^a^ ± 0.132	6.16 ^ab^ ± 0.080	11.9 ^a^ ± 0.315	18.1 ^a^ ± 0.307	22.5 ^a^ ± 1.238
B.U.T. 6 non-trimmed	V2	62.88 ^a^ ± 4.11	0.38 ^a^ ± 0.031	1.93 ^a^ ± 0.164	6.30 ^a^ ± 0.119	11.9 ^a^ ± 0.178	17.7 ^a^ ± 0.277	22.4 ^a^ ± 1.237
B.U.T. Premium trimmed	V3	62.23 ^ab^ ± 4.18	0.36 ^b^ ± 0.030	1.79 ^b^ ± 0.164	5.92 ^c^ ± 0.089	11.3 ^a^ ± 0.329	17.5 ^a^ ± 0.498	21.7 ^b^ ± 1.613
B.U.T. Premium non-trimmed	V4	61.42 ^ab^ ± 4.31	0.34 ^c^ ± 0.034	1.74 ^b^ ± 0.168	5.94 ^bc^ ± 0.112	11.6 ^a^ ± 0.568	17.8 ^a^ ± 0.794	21.6 ^b^ ± 1.326
Auburn trimmed	V5	59.77 ^ab^ ± 4.36	0.31 ^d^ ± 0.023	1.45 ^c^ ± 0.107	4.66 ^d^ ± 0.103	8.63 ^b^ ± 0.231	12.7 ^b^ ± 0.433	15.4 ^c^ ± 0.996
Auburn non-trimmed	V6	59.23 ^b^ ± 3.23	0.32 ^d^ ± 0.019	1.49 ^c^ ± 0.106	4.64 ^d^ ± 0.106	8.41 ^b^ ± 0.225	12.7 ^b^ ± 0.604	15.2 ^c^ ± 0.869

P = phase; V = variant; LW = week of life; B.U.T. = British United Turkey; different superscript lowercase letters within a column indicate a main effect (*p* ≤ 0.05).

**Table 3:** Statistical analysis of the relationship between the parameters age, lux (light intensity) and NH_3_ (gaseous ammonia content) and the examined welfare indicators (*n* = 192). Shown with the odds ratio, the standard error in brackets and the 95% credible interval below. Significant differences are marked with an asterisk.

Parameter				Dependent Variable		
	Injury on the Body	Injury on the Snood	Plumage on the Tail Feathers	Breast Buttons	Food Pad Dermatitis	Beak State
Age	1.04 (0.01) * 1.03–1.05	1.03 (0.01) * 1.02–1.04	1.03 (0.01) * 1.02–1.05	1.06 (0.01) * 1.04–1.08	1.04 (0.01) * 1.04–1.05	1.03 (0.01) * 1.02–1.04
Lux	1.01 (0.01) 0.98–1.04	0.99 (0.01) 0.97–1.01	1.01 (0.02) 0.98–1.05	1.05 (0.03) * 1.01–1.11	0.98 (0.01) 0.95–1.00	0.97 (0.03) 0.90–1.03
NH_3_	0.77 (0.34) 0.29–1.61	1.41 (0.55) 0.60–2.71	0.62 (0.52) 0.10–1.99	0.19 (0.19) * 0.02–0.71	0.82 (0.32) 0.36–1.59	0.89 (0.69) 0.19–2.71

**Table 4:** Statistical analysis of the examined welfare indicators with the contrasts between the three breeds and the beak-trimmed and non-beak-trimmed turkeys (*n* = 192). Shown with the odds ratio, the standard error in brackets and the 95% credible interval below. This analysis was calculated without the variables age, lux (light intensity), and NH_3_ (ammonia content). Significant differences are marked with an asterisk.

Beak Treatment or Breed	Contrast	Dependent Variable					
		Injury on the Body	Injury on the Snood	Plumage on the Tail Feathers	Breast Buttons	Food Pad Dermatitis	Beak State
non-trimmed	B.U.T. Premium vs. B.U.T. 6	2.50 (1.52) 0.80–6.36	1.73 (0.56) 0.90–3.06	0.23 (0.15) * 0.06–0.59	0.90 (0.72) 0.19–2.55	0.50 (0.22) 0.20–1.03	3.03 (11.68) 0.15–11.58
	Auburn vs. B.U.T. 6	0.50 (0.29) 0.16–1.22	0.23 (0.08) * 0.11–0.42	0.04 (0.04) * 0–0.15	0.06 (0.06) * 0.01–0.20	2.70 (1.18) * 1.12–5.61	1.42 (7.67) 0.08–5.87
	B.U.T. Premium vs. Auburn	5.75 (3.59) * 1.78–15.01	7.87 (2.72) * 3.99–14.55	53.81 (745.87) * 1.08–230.20	29.88 (106.45) * 3.62–122.08	0.20 (0.09) * 0.08–0.41	4.38 (11.13) 0.23–17.51
trimmed	B.U.T. Premium vs. B.U.T. 6	1.73 (0.99) 0.53–4.16	1.42 (0.46) 0.72–2.50	0.38 (0.21) * 0.12–0.90	1.02 (1.03) 0.19–3.32	0.76 (0.36) 0.30–1.64	n.m.
	Auburn vs. B.U.T. 6	0.30 (0.19) * 0.09–0.77	0.51 (0.17) * 0.25–0.91	0.03 (0.04) * 0–0.14	0.05 (0.06) * 0–0.18	7.98 (3.69) * 3.22–16.97	n.m
	B.U.T. Premium vs. Auburn	6.72 (4.04) * 1.94–16.89	2.96 (1.01) * 1.46–5.35	122.92 (5215.61) * 2.13–403.26	70.37 (517.49) * 3.78–359.21	0.1 (0.05) * 0.04–0.22	n.m.
B.U.T. 6	trimmed vs. non-trimmed	0.68 (0.4) 0.20–1.64	0.54 (0.18) * 0.28–0.95	1.17 (0.67) 0.42–2.65	0.75 (0.85) 0.14–2.13	0.50 (0.23) 0.20–1.05	n.m.
B.U.T. Premium	trimmed vs. non-trimmed	0.47 (0.25) 0.13–1.10	0.44 (0.14) * 0.22–0.76	2.25 (1.77) 0.53–6.64	0.81 (0.85) 0.16–2.39	0.76 (0.35) 0.30–1.61	n.m.
Auburn	trimmed vs. non-trimmed	0.41 (0.25) 0.12–1.03	1.18 (0.41) 0.57–2.16	8.59 (122.11) 0.03–36.48	0.78 (1.66) 0.04–3.56	1.47 (0.63) 0.61–3.03	n.m.

B.U.T. = British United Turkey; vs. = versus; n.m.: not measured because beak-trimmed turkeys had Score 0 in the variable beak state.

**Table 5:** Rating of the examined animal welfare indicators in percentage (%) of turkeys (*n* = 192) with alterations or injuries (> Sore 0) on the assessed body parts after Phase 6 (137th DOL).

Breed and Beak Treatment	Plumage on the Back and Wings	Injury on the Body	Injury on the Snood	Plumage on the Tail Feathers	Breast Blister	Breast Button	Foot Pad Dermatitis
B.U.T. 6 trimmed	0.6 ^a^	34.4 ^a^	35.6 ^b^	15.0 ^a^	0.0 ^a^	16.2 ^a^	35.6 ^b^
B.U.T. 6 non-trimmed	0.0 ^a^	44.4 ^a^	48.2 ^a^	14.4 ^a^	0.0 ^a^	18.1 ^a^	50.0 ^b^
B.U.T. Premium trimmed	0.0 ^a^	41.5 ^a^	41.2 ^b^	6.3 ^b^	0.6 ^a^	10.1 ^a^	28.9 ^b^
B.U.T. Premium non-trimmed	1.2 ^a^	54.0 ^ab^	56.3 ^a^	3.7 ^b^	0.6 ^a^	15.5 ^a^	35.4 ^b^
Auburn trimmed	0.0 ^a^	15.6 ^c^	22.9 ^c^	0.6 ^c^	0.0 ^a^	0.2 ^b^	75.6 ^a^
Auburn non-trimmed	0.0 ^a^	29.4 ^bc^	21.2 ^c^	0.6 ^c^	0.0 ^a^	2.5 ^b^	68.8 ^a^

B.U.T. = British United Turkey; different superscript lowercase letters within a column indicate a main effect (*p* ≤ 0.05).

**Table 6:** Beak measurements and the standard deviation (±) in millimeters (mm) at the end of Phase 6 (137th DOL) (*n* = 523) in relation to the variant. The length of the upper beak in millimeters was measured from the anterior edge of the nares to the tip of the beak.

Breed and Beak Treatment	Variant	Length of the Upper Beak (mm)	Overlap of the Upper Beak (mm)	Overlap of the Lower Beak (mm)
B.U.T. 6 trimmed	V1	21.2 ^c^ ± 1.08	n/a	4.21 ^ab^ ± 1.92
B.U.T. 6 non-trimmed	V2	31.3 ^a^ ± 1.93	2.24 ^b^ ± 1.67	n/a
B.U.T. Premium trimmed	V3	21.2 ^c^ ± 1.91	n/a	3.99 ^b^ ± 2.08
B.U.T. Premium non-trimmed	V4	30.0 ^b^ ± 1.57	1.44 ^c^ ± 1.73	n/a
Auburn trimmed	V5	19.7 ^d^ ± 1.13	n/a	4.42 ^a^ ± 1.62
Auburn non-trimmed	V6	30.0 ^b^ ± 2.18	2.68 ^a^ ± 1.71	n/a

B.U.T. = British United Turkey; V = variant; different superscript lowercase letters within a column indicate a main effect (*p* ≤ 0.05); n/a = not applicable.

**Table 7:** Beak measurements and the standard deviation (±) in millimeters (mm) of the slaughtered turkeys after Phase 5 (118th DOL) (*n* = 48) in relation to the variant.

Breed and Beak Treatment	Variant	Length of the Upper Beak (mm)	Overlap of the Upper Beak (mm)	Horny Layer Tip (Upper Beak, mm)
B.U.T. 6 trimmed	V1	21.5 ^b^ ± 1.20	n/a	2.10 ^b^ ± 0.65
B.U.T. 6 non-trimmed	V2	29.1 ^a^ ± 1.13	1.60 ^b^ ± 0.79	2.48 ^ab^ ± 0.48
B.U.T. Premium trimmed	V3	20.9 ^bc^ ± 0.64	n/a	0.76 ^c^ ± 0.71
B.U.T. Premium non-trimmed	V4	29.4 ^a^ ± 1.19	1.45 ^b^ ± 1.11	2.27 ^b^ ± 0.92
Auburn trimmed	V5	19.6 ^c^ ± 1.58	n/a	1.24 ^c^ ± 0.26
Auburn non-trimmed	V6	29.6 ^a^ ± 0.92	2.54 ^a^ ± 0.85	3.00 ^a^ ± 0.97

B.U.T. = British United Turkey; V = variant; different superscript lowercase letters within a column indicate a main effect (*p* ≤ 0.05); n/a = not applicable.

**Table 8:** Beak measurements and the standard deviation (±) in millimeters (mm) of the slaughtered turkeys after Phase 6 (138th DOL) (*n* = 48) in relation to the variant.

Breed and Beak Treatment	Variant	Length of the Upper Beak (mm)	Overlap of the Upper Beak (mm)	Horny Layer Tip (Upper Beak, mm)	Overlap of the Lower Beak (mm)
B.U.T. 6 trimmed	V1	21.5 ^c^ ± 0.76	n/a	1.09 ^b^ ± 0.50	4.53 ^a^ ± 1.88
B.U.T. 6 non-trimmed	V2	32.6 ^a^ ± 1.06	3.23 ^a^ ± 0.98	2.50 ^a^ ± 0.94	n/a
B.U.T. Premium trimmed	V3	21.5 ^c^ ± 0.93	n/a	1.21 ^b^ ± 0.67	5.08 ^a^ ± 2.49
B.U.T. Premium non-trimmed	V4	31.4 ^b^ ± 1.30	2.22 ^b^ ± 0.99	3.09 ^a^ ± 0.94	n/a
Auburn trimmed	V5	19.3 ^d^ ± 0.89	n/a	1.33 ^b^ ± 0.31	5.35 ^a^ ± 1.75
Auburn non-trimmed	V6	30.6 ^b^ ± 2.00	1.63 ^b^ ± 1.57	3.27 ^a^ ± 1.37	n/a

B.U.T. = British United Turkey; V = variant; different superscript lowercase letters within a column indicate a main effect (*p* ≤ 0.05); n/a = not applicable.

## Data Availability

The data presented in this study are available on request from the corresponding authors (S.G. and S.B.).

## Appendix A. Ingredients of the Food

**Table A1:** Ingredients of turkey feed. Feed manufracturers: Deutsche Tierernährung Cremer GmbH & Co. Kg, Passauerstraße 11, 93055 Regensburg, Germany; bought at: BayWA AG FM/TH Franken, Werk A143, Lagerhausstraße 26, 96052 Bamberg.

Feed:		Phase (P)	Ingredients:
**Gallugold PMK 1 GRAN 617001025**	granulated	P1	27,5 % Crude protein / 5,2 % Crude fat / 3,8 % Crude fiber / 7,8 % Crude ash / 1,35 % Calcium / 0,9 % Phosphorus / 0,14 % Sodium / 1,75 % Lysine / 0,7 % Methionine calculated as Equivalent of Methionine 11,4 MJ ME/kg
**Bonimal GK PMK 2 PELL 6170820**	pellets	P2	25,5 % Crude protein / 4,1 % Crude fat / 3,1 % Crude fiber / 7,1 % Crude ash / 1,25 % Calcium / 0,83 % Phosphorus / 0,15 % Sodium / 1,6 % Lysine / 0,63 % Methionine calculated as Equivalent of Methionine 11,7 MJ ME/kg
**Bonimal GK PMK 3 OG PELL 6171520**	pellets	P3	23,0 % Crude protein / 6,5 % Crude fat / 3,6 % Crude fiber / 5,8 % Crude ash / 1,1 % Calcium / 0,7 % Phosphorus / 0,16 % Sodium / 1,45 % Lysine / 0,56 % Methionine calculated as Equivalent of Methionine 12,2 MJ ME/kg
**Bonimal GK PMK 4 OG PELL 6172220**	pellets	P4	20,0 % Crude protein / 6,6 % Crude fat / 3,2 % Crude fiber / 5,3 % Crude ash / 0,95 % Calcium / 0,6 % Phosphorus / 0,16 % Sodium / 1,25 % Lysine / 0,5 % Methionine calculated as Equivalent of Methionine 12,5 MJ ME/kg
**Bonimal GK PMK 5 OG PELL 6173020**	pellets	P5	17,0 % Crude protein / 7,0 % Crude fat / 3,2 % Crude fiber / 4,8 % Crude ash / 0,85 % Calcium / 0,55 % Phosphorus / 0,16 % Sodium / 1,15 % Lysine / 0,4 % Methionine calculated as Equivalent of Methionine 12,8 MJ ME/kg
**Bonimal GK PMK 6 OG PELL 6173820**	pellets	P6	15,5 % Crude protein / 7,4 % Crude fat / 3,1 % Crude fiber / 4,4 % Crude ash / 0,8 % Calcium / 0,5 % Phosphorus / 0,17 % Sodium / 1,05 % Lysine / 0,39 % Methionine calculated as Equivalent of Methionine 13,2 MJ ME/kg

## Appendix B. Scoring Systems

**Table A2:** Scoring system of the plumage of the back and wings [25].

Score	Definition
**0**	no featherless areas
**1**	featherless areas < 5cm
**2**	featherless areas 5cm–10cm
**3**	featherless areas > 10cm

**Table A3:** Scoring system of the plumage of the tail [25].

Score	Definition
**0**	tail mostly intact
**1**	in the majority of the tail feathers only quills are left, the majority of the tips of the tail feathers are missing
**2**	tail feathers shortened by 50%
**3**	tail feathers severily shortened or completely missing

**Table A4:** Scoring system of the injuries on the body [25].

Score	Definition
**0**	no visible changes (including hematoma and scratches)
**1**	injuries (including hematoma and scratches) < 2cm
**2**	injuries (including hematoma and scratches) 2cm–8 cm
**3**	injuries (including hematoma and scratches) > 8cm

**Table A5:** Scoring system of the injuries on the snood [25].

Score	Definition
**0**	no visible changes
**1**	≤ 50% of the skin of the snood is scabbed
**2**	> 50% of the skin of the snood is scabbed
**3**	100% of the skin of the snood is scabbed or the snood is clearly shortened / missing

**Table A6:** Scoring of the breast skin [26].

Score	Breast Blister	Breast Button
**0**	no breast blister	no breast button
**1**	slightly fluctuating, no or small elevation	in formation, small induration recognizable
**2**	fist-sized, fluctuating or hardened elevation	breast button 1cm–2cm
**3**	double-fist-sized fluctuating or hardened elevation	breast button > 2.5cm

**Table A7:** Scoring of the foot pat health [27].

Score	Definition
**0**	foot pads completely intact
**1**	superficial signs of wear and tear, or small, punctiform, darkly discolored areas (necroses) on the metatarsus with no swelling of the foot pads, the toes are not affected.
**2**	darkly discolored areas (necroses), affecting a maximum of ¼ of the foot pads, slight swelling of the foot pads, toes are not affected
**3**	darkly discolored areas (necroses), affecting ¼ to ½ of the foot pads, swelling of the foot pads or scabby, crusty deposits, toes could be affected
**4**	darkly discolored areas (necroses), affecting more than ½ of the foot pads or very strong swelling of the foot pads, or scrabby, crusty deposits with deep injuries, toes are also affected

**Table A8:** Scoring of the beak [15].

Score	Definition
**0**	infrared beak trimmed
**1**	no abrasion, overlap of the upper beak
**2**	medium abrasion, a more slightly overlap of the upper beak
**3**	great abrasion, upper- and lower beak have mainly the same length
